# The Effectiveness of Acupuncture for Dysphagia after Stroke: A Systematic Review and Meta-Analysis

**DOI:** 10.1155/2021/8837625

**Published:** 2021-01-19

**Authors:** Lida Zhong, Jing Wang, Fang Li, Xiao Bao, Huiyu Liu, Pu Wang

**Affiliations:** ^1^Department of Rehabilitation Medicine, Yuebei People's Hospital, Shaoguan, Guangdong 512026, China; ^2^Department of Rehabilitation Medicine, The Seventh Affiliated Hospital Sun Yat-sen University, Shenzhen, Guangdong 518107, China

## Abstract

**Objectives:**

This study reviewed and evaluated existing evidence of the efficacy of acupuncture as a clinical treatment for dysphagia after stroke.

**Methods:**

Five English and four Chinese databases were searched from inception to March 2020. All randomized controlled trials (RCTs) incorporating acupuncture or acupuncture combined with other interventions for the treatment of dysphagia after stroke were enrolled. All data were independently assessed and extracted by two authors. The bias risk assessment recommended by the Cochrane Collaboration's tool was used to assess the quality of the selected studies. This meta-analysis was conducted by using RevMan 5.3. Pooled analyses were calculated by the mean difference (MD) and 95% confidence interval (CI). Heterogeneity was assessed by the *I*^2^ test.

**Results:**

Thirty-five studies involving 3024 patients were analyzed. The meta-analysis showed that the therapeutic efficacy of acupuncture combined with other interventions was better than that of the control group for the standardized swallowing assessment (SSA) score (MD = −3.78, 95% CI: −4.64 to −2.91, *P* < 0.00001), Ichiro Fujishima rating scale (IFRS) score (MD = 1.68, 95% CI: 1.16 to 2.20, *P* < 0.00001), videofluoroscopic swallowing study (VFSS) score (MD = 2.26, 95% CI: 1.77 to 2.74, *P* < 0.00001), and water swallowing test (WST) score (MD = −1.21, 95% CI: −1.85 to −0.57, *P*= 0.0002). In studies reporting adverse effects, no serious outcome from an adverse event was confirmed.

**Conclusion:**

This systematic review indicated that acupuncture could be an effective therapy for treating dysphagia after stroke although stricter evaluation standards and rigorously designed RCTs are needed.

## 1. Introduction

Dysphagia is one of the most common poststroke sequelae, accounting for 27 to 64% of stroke patients [1], and is often associated with malnutrition, pneumonia, and dehydration [2]. The previous study [3] has shown that dysphagia after stroke affects quality of life, carries increased risks of mortality and dependency, prolongs hospital stays, increases healthcare costs, and often leads to discharge from the hospital to a care home. Therefore, to accelerate the recovery of swallowing function and reduce these risks, it is very important to find an effective treatment for dysphagia.

At present, there are many treatments for dysphagia, such as behavioral interventions, drug therapy, physical stimulation, and transcranial magnetic stimulation. Some of these treatments have made considerable progress [4]. However, clinical evidence to establish their roles in the management of poststroke dysphagia is limited [4], and there is no clear treatment for dysphagia.

Acupuncture, as a form of alternative medicine, is a traditional treatment that is clinically effective for neurological diseases [5, 6]. Acupuncture treatment exerts therapeutic effects by inserting a needle at specific acupoints on the body surface with stimulation delivery via manual rotation or electric pulses [7–9]. Some randomized controlled trials (RCTs) [10, 11] have shown that acupuncture may reduce the proportion of participants with dysphagia at the end of the trial. However, despite the high heterogeneity, the latest updated Cochrane review [12] on swallowing therapy, which included an analysis of acupuncture, failed to show improvement in swallowing ability. There is still a lack of high-quality research on acupuncture treatment of dysphagia [12], and many clinical studies are still in the preliminary stage, with great differences in the acupuncture methods and the selection of acupoints in the research, leading to the inconclusive conclusion of acupuncture treatment for dysphagia.

This systematic review and meta-analysis aimed to evaluate the potential availability and safety of acupuncture for poststroke dysphagia.

## 2. Methods

The protocol was registered on the International Platform of Registered Systematic Review and Meta-analysis Protocols (INPLASY2020100036), and it was conducted according to the preferred reporting items for systematic reviews and meta-analysis (PRISMA): The PRISMA Statement [13].

### 2.1. Search Strategy

We searched the following databases from their inception until March 2020: EMBASE (via Ovid), MEDLINE (via Ovid), the Cochrane library (via Ovid), PubMed (via website), ScienceDirect (via website), China National Knowledge Infrastructure (CNKI) (via website), China Biology Medicine disc (CBMdisc) (via website), China Science and Technology Journal Database (VIP) (via website), and Wanfang Data (via website). Manual searches of relevant references were also conducted. The search terms were (“dysphagia,” “swallowing disorders,” “deglutition disorders,” or “swallowing dysfunction”) and (“stroke,” “cerebral apoplexy,” or “cerebrovascular accident”) and (“acupuncture,” “needling,” “electroacupuncture,” or “warm acupuncture”).

### 2.2. Inclusion and Exclusion Criteria

#### 2.2.1. Types of Studies

All RCTs of acupuncture for dysphagia after stroke were selected and excluded nonrandomized studies, observational studies, animal studies, qualitative studies, and letters.

#### 2.2.2. Types of Participants

All patients conformed to the explicit clinical diagnosis criteria of stroke and dysphagia: (1) the participants were clinically diagnosed with ischemic or hemorrhagic stroke by computerized tomography or magnetic resonance imaging; (2) dysphagia was diagnosed using a clinical bedside swallowing assessment, a videofluoroscopic swallowing study (VFSS), or a fiberoptic endoscopic examination of swallowing (FEES).

#### 2.2.3. Types of Interventions

For the intervention in experimental trials, acupuncture alone or acupuncture combined with other interventions was included, and other interventions included behavioral interventions, drug therapy, and electrical stimulation. The interventions should be the same between experimental and control trials, except for acupuncture in the experimental trials.

#### 2.2.4. Types of Outcome Measures

The clinical symptoms had obviously improved with specific evaluation standards, such as (1) Watian swallowing test (WST) [14], (2) standardized swallowing assessment (SSA) [15–17], (3) penetration-aspiration scale (PAS) [18], and (4) functional oral intake scale (FOIS) [19], or by using an objective index, such as (1) VFSS [20] and (2) endoscopic evaluation of swallowing [21], as the efficacy evaluation criterion.

### 2.3. Data Extraction

Data were extracted by three review authors (Lida Zhong, Jing Wang, and Fang Li) independently using a standardized form after evaluation. Disagreements were resolved with the assistance from a fourth author (Pu Wang), if necessary. Data extracted included the surname of the first author, year of publication, intervention used in the acupuncture and control groups, evaluation time, outcomes, conclusions, follow-up duration, and adverse effects.

### 2.4. Risk of Bias Assessment

The included RCTs were assessed according to the Cochrane risk of bias assessment tool [22], and this process was carried out independently by the two review authors (Lida Zhong and Jing Wang). Quality was assessed as having a low, an unclear, or a high risk of bias according to seven criteria: (1) random allocation method (selection bias); (2) allocation concealment (selection bias); (3) blinding of assessors (performance bias); (4) blinding of outcome assessment (detection bias); (5) integrity of data results (attrition bias); (6) selective reporting (reporting bias); and (7) other sources of bias. Any disagreements that arouse at any stage between the two reviews were resolved through discussion with a third author (Pu Wang).

### 2.5. Statistical Analysis

All statistical analyses were performed using RevMan 5.3 (http://ims.cochrane.org/revman). For dichotomous variables, the relative risk (RR) with its 95% confidence interval (CI) was calculated. For continuous variables, the mean difference (MD) and standardized mean difference (SMD) with their 95% CIs were calculated. The heterogeneity between each group was tested by Cochran's *Q* statistic and the *I*^2^ test [23]. Studies with an *I*^2^ of 25% to 50% were considered to have low heterogeneity, and *I*^2^ values of 50% to 75% and >75% were considered indicative of moderate and high levels of heterogeneity, respectively. Fixed-effect models were used to combine studies if the *I*^2^ test was not significant (*P* for heterogeneity<0.1). Otherwise, random-effect models were used. *P* < 0.05 was considered statistically significant for the between-group difference. If substantial heterogeneity was detected, we looked for reasonable explanations, and subgroup analysis or sensitivity analysis could be applied to explore the causes of heterogeneity. If the sources of heterogeneity could not be determined, a descriptive analysis was adopted.

## 3. Results

### 3.1. Study Selection

**Figure 1:**
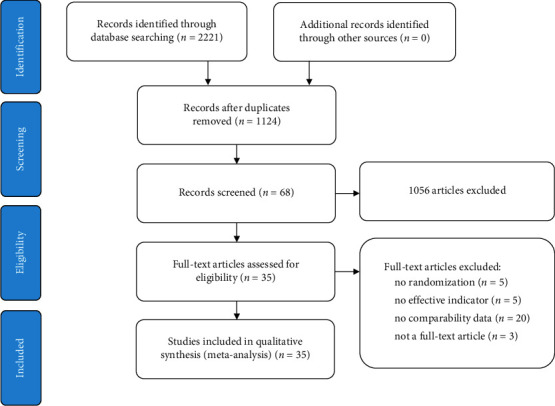
Flow diagram for the selection of the included studies.

The PRISMA flow diagram of the literature search and the results are shown in Figure 1. These studies were screened for eligibility using the detailed participant, intervention, comparison, and outcome (PICO) criteria. The initial search of computerized databases retrieved a total of 2221 articles. After removing duplicates, 1124 articles were found, of which 68 records were subjected to a full-text review. We excluded 33 articles for the following reasons: no randomization (*n* = 5), no effective indicator (*n* = 5), no comparability data (*n* = 20), and not a full-text article (*n* = 3). Finally, 35 RCTs were included in this review.

### 3.2. Description of Studies

The characteristics of the included studies in this review are shown in Table 1. Among the 35 included studies, all the studies were conducted in China. Nine of thirty-five articles [24, 26–28, 31, 32, 35, 38, 41] were reported in an English database, and the remaining were reported in a Chinese database. Overall, 35 eligible studies involved 3024 participants diagnosed with dysphagia after stroke, and they were published between 2006 and 2020. All trials compared acupuncture with a swallowing treatment. In these trials, the frequency of acupuncture intervention was at least three times a week for more than two weeks in duration. Three studies [28, 32, 49] reported adverse events, and four studies [25, 28, 31, 40] reported dropouts.

### 3.3. Assessment for Risk of Bias

**Figure 2:**
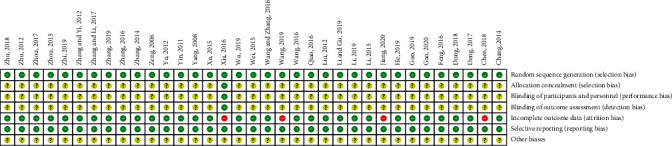
Potential risk of bias of each included study.

**Figure 3:**
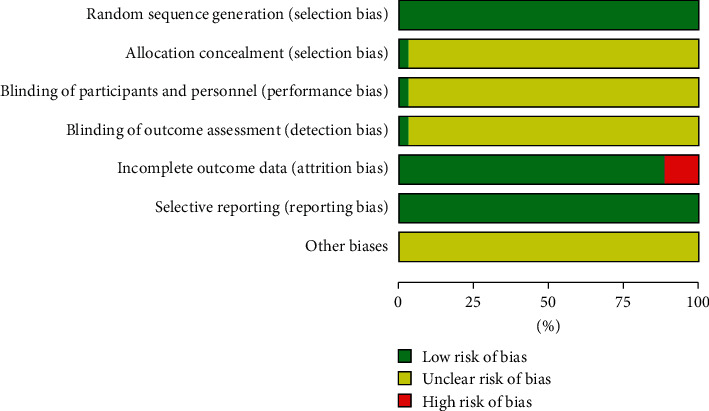
Summary of bias evaluation for the studies.

The details of the overall risk of bias across the 35 RCTs are provided in Table 2. Of the 35 included studies, the randomization procedure was reported in adequate detail in all studies. One trial [28] clearly reported the allocation concealment, the blinding of participants and personnel, and the blinding of outcome assessment; other descriptions in the other studies were unclear. Four trials [25, 28, 31, 40] excluded dropout participants for the data analysis, which may increase the risk of attrition bias. All the studies clearly described the selective reporting. In total, 4 out of 35 studies (11.43%) were judged as having a high risk of bias because one of the main aspects of the bias assessments was high (Figures 2 and 3).

### 3.4. Standard Swallowing Assessment (SSA)

**Figure 4:**
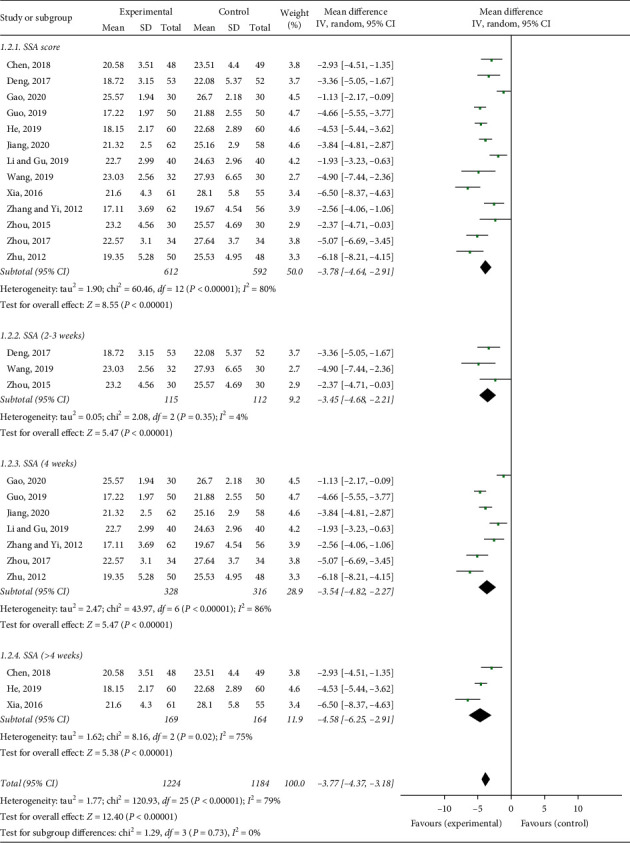
Forest plot of the SSA effective rate.

There were 13 studies that used the SSA as the effective evaluation standard with continuous data. The meta-analysis showed a MD with high heterogeneity (*I*^2^ = 80%). Therefore, the random-effect model was used (Figure 4), and we performed a subgroup analysis according to the course of the disease. Heterogeneity was found to remain unaltered although no source for it was identified. Meanwhile, the meta-analysis results showed significant differences in SSA scores in dysphagia between the acupuncture and control groups. The acupuncture group had lower SSA scores than the control group (MD = −3.78, 95% CI: −4.64 to −2.91, *P* < 0.00001) (Figure 4).

### 3.5. Ichiro Fujishima Rating Scale (IFRS)

**Figure 5:**
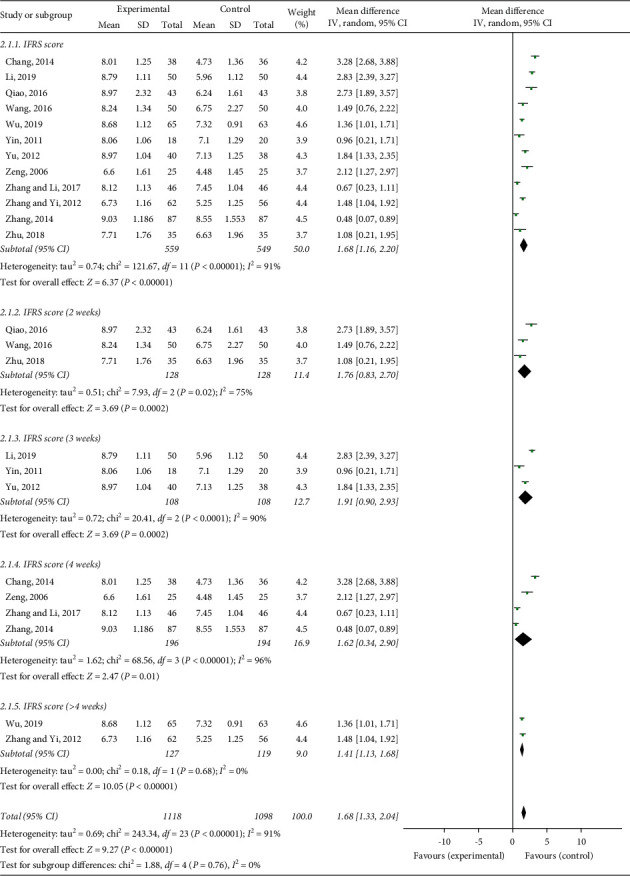
Forest plot of IFRS effective rate.

**Figure 6:**
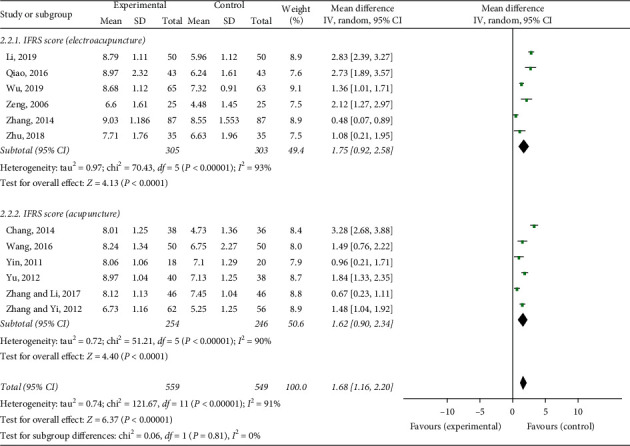
Forest plot of IFRS subgroup analysis.

Twelve studies used the Ichiro Fujishima rating scale as the evaluation standard. The meta-analysis indicated that the acupuncture group had obviously improved IFRS scores (MD = 1.68, 95% CI: 1.16 to 2.20, *P* < 0.00001, *I*^2^ = 91%) (Figure 5). The heterogeneity was high, and we found that the treatment durations and the acupuncture methods were different in these 12 studies, so we performed a subgroup analysis. Subgroup analysis of six articles seemed to show that electroacupuncture combined with swallowing treatment was more effective than swallowing treatment alone (MD = 1.75, 95% CI: 0.92 to 2.58, *P* < 0.00001) (Figure 6). The subgroup analysis of these six articles seemed to show that simple acupuncture combined with swallowing treatment was more effective than swallowing treatment alone (MD = 1.62, 95% CI: 0.90 to 2.34, *P* < 0.00001) (Figure 6). However, we did not find a clear source of heterogeneity for IFRS with an *I*^2^ statistic that ranged from 75% to 96% in subgroup analyses, such as different acupuncture stimulation parameters, different acupoint selections, different needle holding times, and treatment durations.

### 3.6. Videofluoroscopy (VFSS)

**Figure 7:**
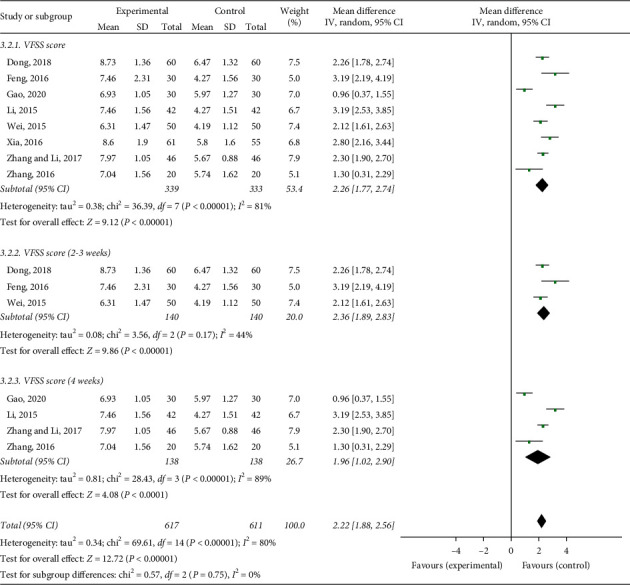
Forest plot of the VFSS effective rate.

Among the included studies, 8 used videofluoroscopy to evaluate the effectiveness of the treatment with continuous data. The result exhibited a MD with medium heterogeneity (*I*^2^ = 81%). We performed a subgroup analysis, and heterogeneity was found to remain unaltered although no source for it was identified. The meta-analysis showed that acupuncture combined with swallowing treatment produced a sustained and significant improvement, as reflected in the VFSS scores in these stroke patients (MD = 2.26, 95% CI: 1.77 to 2.74, *P* < 0.00001) (Figure 7).

### 3.7. Watian Swallowing Test (WST)

**Figure 8:**
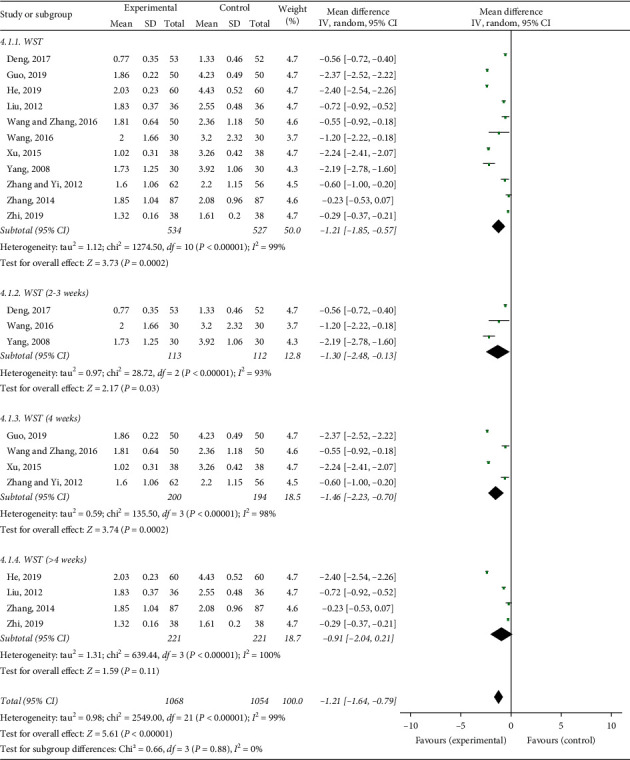
Forest plot of the WST effective rate.

Eleven studies selected the Watian swallowing test as the evaluation standard. The meta-analysis showed a MD with high heterogeneity (*I*^2^ = 99%). We did not find a clear source of heterogeneity for WST with an *I*^2^ statistic that ranged from 93% to 100% in subgroup analyses, such as different acupuncture stimulation parameters, different acupoint selections, different needle holding times, and treatment durations. We could see from the figure that the score of the control group was higher than that of the acupuncture group (Figure 8). This illustrated that the acupuncture group was able to lower the WST scores (MD = −1.21, 95% CI: −1.85 to −0.57, *P*=0.0002) (Figure 8).

### 3.8. Acupuncture Point

**Figure 9:**
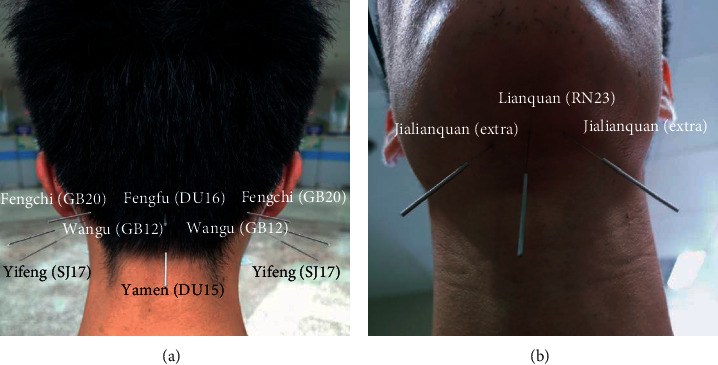
Acupoints in the neck: (a) bilateral Fengchi (GB20), bilateral Wangu (GB12), bilateral Yifeng (SJ17), Fengfu (DU16), and Yamen (DU15); (b) Lianquan (RN23) and bilateral Jialianquan (extra).

**Figure 10:**
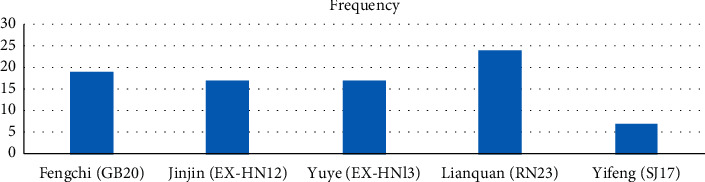
The most frequently used acupoints in these studies.

The selection of acupoints was chosen mainly based on the symptoms and syndrome differentiation of traditional Chinese medicine (TCM) (Figure 9). After analysis of points adopted in these trials, we found that Fengchi (GB20), Jinjin (EX-HN12), Yuye (EX-HNl3), Lianquan (RN23), and Yifeng (SJ17) were the five points most commonly used (Figure 10).

### 3.9. Publication Bias

**Figure 11:**
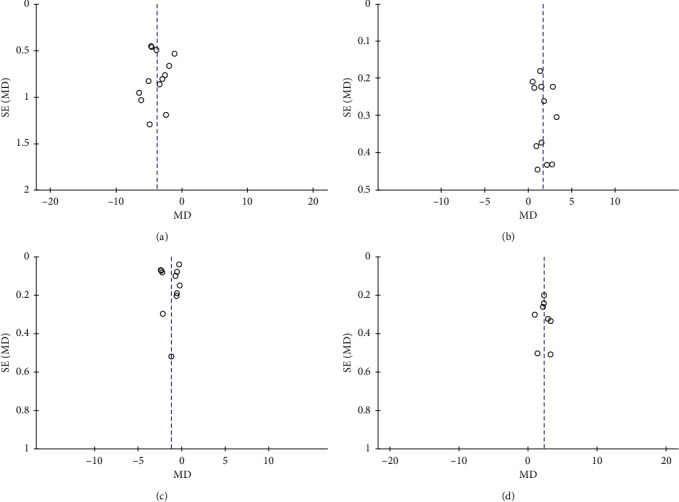
Funnel plot of the publication bias of acupuncture in the (a) SSA, (b) IFRS, (c) VFSS, and (d) WST.

Publication bias was reported via a funnel plot (Figure 11), in which the asymmetry of the funnel plots may have arisen through heterogeneity.

### 3.10. Adverse Events

Three studies reported adverse events [28, 32, 49], while the remaining 32 studies did not mention adverse events. Among the 3 studies, they reported the occurrence of adverse events such as bleeding, pain, and discomfort. However, no life-threatening adverse events were noted in any of the included studies.

## 4. Discussion

The object of this systematic review was to evaluate the effectiveness of acupuncture in treating dysphagia after stroke. This systematic review showed that the therapeutic efficacy of acupuncture combined with other interventions was better than that of the control group in the SSA score, IFRS score, VFSS score, and WST score. In the subgroup analysis, we obtained similar results that acupuncture had a significant effect on dysphagia.

The advantage of this systematic review was that most of the included RCTs had a low or moderate risk of bias. However, we acknowledge that there are some limitations in this review. First, a language bias may exist because all of the included trials were conducted and published by Chinese investigators. Second, in the present study, only one trial [28] reported allocation concealment, blinding of performance, and blinding of assessment; these risks of bias may affect the interpretation of the results. Finally, four trials [25, 28, 31, 40] excluded dropout participants for data analysis, which may increase the risk of attrition bias.

Poststroke dysphagia is in the category of “unsound speech and motor impairment” in TCM. It is believed in TCM that the etiology and pathogenesis of dysphagia are pathogenic wind, fire, phlegm, blood stasis, and qi deficiency, leading to the dysfunction of Zang-fu organs, reverse flow of qi and blood, obstruction of meridians and collaterals by blood stasis, and oppression of the brain marrow. The location of sickness is related to the brain, mouth, tongue, and throat. Hence, acupuncture can be used at the corresponding points to nourish yin, activate collaterals, wake up the brain, open the aperture, and remove obstruction. Some studies [59–61] showed that acupuncture therapy could improve the blood circulation in the cerebral cortex motor functional areas, promote the recovery of central nervous system function, improve the brain energy metabolism, activate the specific motor functional areas of the cerebral cortex, and promote the remodeling of brain function. This may be the main mechanism of acupuncture in treating dysphagia after stroke.

Meta-analyses showed that acupuncture could improve swallowing at different stages of treatment, and some studies [28, 34, 55] showed that the longer the acupuncture treatment lasts, the better the recovery of swallowing function, which may be related to the time it takes to remodel brain functions. However, some studies [38, 48, 51] showed that the recovery of the swallowing function after acupuncture treatment for more than 4 weeks was not as good as that after acupuncture treatment for 4 weeks, which may be related to the different evaluation criteria of the efficacy of swallowing disorders and the diversity of acupuncture treatment options. VFSS and FEES are two instrumental assessments of dysphagia and are considered the “gold standard” for swallowing assessment [62, 63]. However, only 8 studies used VFSS to evaluate the swallowing performance of the participants, and most of the studies used the WST, SSA, and IFRS. These clinical evaluation scales were subjective clinical evaluation tools based on the observation of the evaluator, which may lead to inaccurate evaluation of the treatment effect. Moreover, in the therapeutic schedule of acupuncture, acupoint selection, stimulation method, needle holding time, and treatment durations in the included studies were not identical, which may affect the outcomes. Previous studies [64–66] showed that many factors influenced the efficacy of acupuncture, such as age, comorbidity, gender, disease severity, stimulation of acupuncture, expectations of patients, and doctor-patient interaction, which may be sources of heterogeneity. However, due to the inability to obtain more relevant data, we cannot analyze based on relevant influencing factors. It was necessary to use strict evaluation standards and high-quality RCT designs to explore acupuncture for dysphagia on poststroke.

Electroacupuncture is a technique of acupuncture based on a traditional acupuncture method combined with modern electrotherapy. Six of the 35 studies included in the meta-analysis used electroacupuncture. The results of subgroup analysis showed that both electroacupuncture and simple acupuncture could improve patients' swallowing function, but the effect was not significantly different between the two, which may be related to the small sample size, the diversity of acupuncture treatment options, and the different evaluation criteria for the efficacy of swallowing disorders. There is a need for more high-quality trials with large sample sizes to investigate electroacupuncture.

In this systematic review, the acupoints used in these 35 RCTs were different. Many studies employed individualized acupoints, but there were 5 points that were most commonly used. In this study, the acupoints of the nape were selected according to the adjacent therapeutic effects of the acupoints (Figure 9). Among them, Fengchi (GB 20), an important point for wind, can be used to suppress yang, extinguish wind, dissolve phlegm and benefit the throat, and clear away heat from the head [38]. Lianquan (RN 23) is an important acupoint mainly for aphasia and deglutition disorder, and it can be used to benefit the pharynx [28]. Jinjin (EX-HN12) and Yuye (EX-HN13) are acupoints for dredging meridians, activating collaterals, and regulating and smoothening qi and blood [55]. Yifeng (SJ17) can be used to open depression winds and benefit pharynges [32]. Hence, the above 5 acupoints could nourish yin, activate the collaterals, wake up the brain, open the aperture, and remove obstruction, thus benefiting dysphagia patients.

In this systematic review, only three studies [28, 32, 49] reported adverse events, including bleeding, pain, and discomfort, and the reactions were tolerable and not serious. The remaining RCTs did not mention any adverse events or side effects. Therefore, acupuncture is safe for dysphagia.

The results of this systematic review show that acupuncture may offer some benefits to patients with dysphagia. However, this review has several limitations. First, we searched only Chinese and English databases, which may cause publication bias. Second, most clinical evaluation scales included in this study were subjective clinical evaluation tools based on the observation of the evaluator, which may lead to an inaccurate evaluation of the treatment effect. Third, in the therapeutic schedule of acupuncture, acupoint selection, stimulation method, needle holding time, and treatment durations in the included studies were not identical, which may affect the outcomes. Fourth, in this systematic review, two studies [28, 51] reported a three-month follow-up, and the remaining RCTs reported only the short-term treatment, so the treatment duration and follow-up time were insufficient to draw conclusions. Fifth, the challenge of blinding arises from the unique nature of acupuncture treatment. Acupuncture treatment involves not only a device but also the acupuncture process and its techniques, such as needle insertion and needle manipulations. It was difficult to achieve true double blinding, which may cause a potential performance bias [67]. In the future, to minimize the ascertainment bias of subjects, implementation of the intervention should be carefully designed to achieve effective blinding of the subjects. The outcome assessor should be blinded to the treatment assignment to reduce detection bias in the study, and the statistician involved in data analysis is usually blinded to group assignments so that the data can be analyzed and interpreted appropriately without bias. These limitations could lead to highly heterogeneous results that prevent us from making a definitive conclusion.

## 5. Conclusion

In conclusion, acupuncture for dysphagia after stroke has therapeutic efficacy and safety. More strict evaluation standards and high-quality RCT designs are necessary for further exploring acupuncture for the treatment of dysphagia after stroke.

## Figures and Tables

**Table 1:** Characteristics of included studies.

Reference	Participants	Intervention	Acupoints	Outcome measures	Main conclusion
Wang et al. [24]	G1 (50): 62.14 ± 12.14 G2 (50): 62.37 ± 5.34	G1: A + ST, G2: ST, F: 3 times/week, D: 4 weeks	GB20, GB12, BL10, RN23, ST5, ST40, EX-HN12, EX-HNl3	WST + IFRS	1 WST (G1 < G2) 2 IFRS (G1 > G2)
Jiang et al. [25]	G1 (65): 60 ± 10 G2 (65): 60 ± 9	G1: A + ST + NEST, G2: ST + NEST, F: 5 times/week, D: 4 weeks	Shesanzhen (extra)	HAMA + HAMD + SSA + sEMG	G1 < G2 in all outcomes
Wu et al. [26]	G1 (65): 44.00 ± 2.92 G2 (63): 44.35 ± 2.60	G1: A + ST, G2: ST, F: 5 times/week, D: 6 weeks	EX-HN1, GV20, EX-HN5, GB20, CV23	IFRS	IFRS (G1 > G2)
Wei et al. (2015) [27]	G1 (50): 61.50 ± 4.2 G2 (50): 62.50 ± 4.90	G1: A + ST, G2: ST, F: 7 times/week, D: 2 weeks	RN23, RN22, EX-HN12, EX-HNl3, LI4, PC6, ST36	VFSS + MBI + FIM	G1 > G2 in all outcomes
Xia et al. [28]	G1 (67): 67±9 G2 (63): 66 ± 10	G1: A + ST, G2: ST, F: 6 times/week, D: 6 weeks	PC6, DU26, SP6, HT1, LU5, BL40, GV20, GB20, CV23, Jialianquan (extra), EX-HN12, EX-HNl3	VFSS + SSA + BI + SWAL-QOL	1 SSA (G1 < G2) 2 G1 > G2 in other outcomes
Zeng et al. [29]	G1 (25): 58.01 ± 10.74 G2 (25): 57.98 ± 11.82	G1: A + ST, G2: ST, F: 7 times/week, D: 4 weeks	SJ17, GB12, Ex-HN14, SI17, RN22, ST9, Toupizhen (extra), RN23, EX-HN12, EX-HNl3, Tunyan (extra), Tiyan (extra), ST4, ST6, DU26, RN24	IFRS	IFRS (G1 > G2)
Chang et al. [30]	G1 (38): 46 ± 10 G2 (36): 44 ± 11	G1: A + BT + NEST, G2: BT + NEST, F: 6 times/week, D: 4 weeks	GV20, RN23, EX-HN12, EX-HNl3, Toupizhen (extra)	IFRS	IFRS (G1 > G2)
Chen et al. [31]	G1 (50): 67 ± 11 G2 (50): 67 ± 10	G1: A + ST, G2: ST, F: 6 times/week, D: 8 weeks	GB20, Ex-HN14, Gongxue (extra), Zhiqiang (extra), Tunyan (extra), Fayin (extra), RN23, EX-HN12, EX-HNl3	RSST + WST + SSA	1 WST (G1 < G2) 2 RSST (G1 > G2) 3 SSA (G1 < G2)
Feng et al. [32]	G1 (30): 60 ± 12 G2 (30): 58 ± 12	G1: A + ST, G2: ST, F: 7 times/week, D:3 weeks	RN23, SJ17, GB12, GB20, DU16, DU15, ST5, EX-HN12, EX-HNl3, Shezhen (extra)	VFSS + WST	1 WST (G1 < G2) 2 VFSS (G1 > G2)
Guo et al. [33]	G1 (50): 66.21 ± 8.03 G2 (50): 65.91 ± 7.85	G1: A + ST, G2: ST, F: 6 times/week, D: 4 weeks	DU15, DU16, BL10, Zhiqiang (extra), RN23	SSA + WST + BI	1 WST (G1 < G2) 2 BI (G1 > G2) 3 SSA (G1 < G2)
He et al. [34]	G1 (60): 62.16 ± 7.04 G2 (60): 61.83 ± 6.81	G1: A + ST, G2: ST, F: 5 times/week, D: 8 weeks	GB20, SJ17, RN23	SSA + WST + BI	1 WST (G1 < G2) 2 BI (G1 > G2) 3 SSA (G1 < G2)
Li et al. [35]	G1 (42): 57.4 ± 4.8 G2 (42): 57.4 ± 4.8	G1: A + BT + ST, G2: BT + ST, F: 6 times/week, D: 4 weeks	Shezhen (extra)	VFSS	VFSS (G1 > G2)
Li et al. [36]	G1 (50): 42.6 ± 2.3 G2 (50): 42.5 ± 2.2	G1: A + ST, G2: ST, F: 7 times/week, D: 3 weeks	GB20, Ex-HN14, Gongxue (extra), Zhiqiang (extra), Tunyan (extra) RN23, EX-HN12, EX-HNl3	IFRS + WST	1 WST (G1 < G2) 2 IFRS (G1 > G2)
Li and Gu [37]	G1 (40): 61.9 ± 7.9 G2 (40): 63.6 ± 6.9	G1: A + BT + ST, G2: BT + ST, F: 6 times/week, D: 4 weeks	GB20, DU16, HT5, LR3	PAS + SSA + WST	G1 < G2 in all outcomes
Liu et al. [38]	G1 (36): 57.6 ± 8.2 G2 (36): 58.5 ± 8.7	G1: A + BT, G2: BT, F: 6 times/week, D: 8 weeks	GB20, Ex-HN14, CV23, Ex-HN12, Ex-HN13, SP6, LR3, ST40, LI4	WST	WST (G1 < G2)
Qiao et al. [39]	G1 (43): 52.27 ± 10.45 G2 (43): 52.86 ± 10.72	G1: A + BT + ST, G2: BT + ST, F: 3 times/week, D: 2 weeks	GB20, DU16, EX-HN15, RN23, Jialianquan (extra), EX-HN12, EX-HNl3, LI4, LR3, HT5	IFRS + WST	1 WST (G1 < 2) 2 IFRS (G1 > G2)
Wang et al. [40]	G1 (35): 64 + 8 G2 (35): 65 ± 9	G1: A + ST, G2: ST, F: 5 times/week, D: 3 weeks	Shesanzhen (extra)	SSA + WST + SWAL-QOL	1 WST (G1 < G2) 2 SWAL-QOL (G1 > G2) 3 SSA (G1 < G2)
Wang [41]	G1 (30): 57.6 ± 9.1 G2 (30): 59.6 ± 8.9	G1: A + BT + NEST, G2: BT + NEST, F: 7 times/week, D: 2 weeks	RN23, Jialianquan (extra), HT5, DU20, Zuqianjin (extra), Zuwujin (extra)	WST	WST (G1 < G2)
Xu [42]	G1 (38): 62.74 ± 5.19 G2 (38): 63.19 ± 4.38	G1: A + BT, G2: BT, F: 5 times/week, D: 4 weeks	GB20, BL10, DU16, RN23, EX-HN12, EX-HNl3, PC6, HT5, SP6, ST36, RN12, BL23, SP3, KI3	WST	WST (G1 < G2)
Yang et al. [43]	G1 (30): 65.8 G2 (30): 67.3	G1: A + BT, G2: BT, F: 5 times/week, D: 2 weeks	GV20, SJ17, RN23, SP6, ST36	WST	WST (G1 < G2)
Yu et al. [44]	G1 (40): 63 ± 10 G2 (38): 64 ± 11	G1: A + ST, G2: ST, F: 7 times/week, D: 3 weeks	DU26, LI4, DU15, DU16, GB20, RN23	IFRS + WST	1 WST (G1 < G2) 2 IFRS (G1 > G2)
Zhu [45]	G1 (50): 65.05 ± 8.99 G2 (48): 64.03 ± 9.83	G1: A + BT + ST, G2: BT + ST, F: 6 times/week, D: 4 weeks	GB20, GB12, SJ17, Shanglianquan (extra), ST9	SSA + SWAL-QOL	1 SWAL-QOL (G1 > G2) 2 SSA (G1 < G2)
Zhou et al. [46]	G1 (34): 59.90 ± 3.87 G2 (34): 60.43 ± 4.07	G1: A + ST + NEST, G2: ST + NEST, F: 6 times/week, D: 4 weeks	Toupizhen (extra)	SSA + SWAL-QOL + CT7R	1 SSA (G1 < G2) 2 G1 > G2 in other outcomes
Zhou et al. [47]	G1 (30): 68.30 ± 13.84 G2 (30): 70.26 ± 11.97	G1: A + ST + NEST, G2: ST + NEST, F: 6 times/week, D: 2 weeks	Toupizhen (extra)	SSA + VFSS + WST + CT7R	1 CT7R (G1 > G2) 2 VFSS (G1 > G2) 3 SSA (G1 < G2) 4 WST (G1 < G2)
Zhi et al. [48]	G1 (39): 63.16 ± 6.92 G2 (39): 62.78 ± 6.78	G1: A + BT + ST, G2: BT + ST, F: 6 times/week, D: 12 weeks	RN23, Jialianquan (extra), EX-HN12, EX-HNl3, Shexiaxue (extra)	WST + GUSS + BI + FMA	1 WST (G1 < G2) 2 G1 > G2 in other outcomes
Zhang and Li [49]	G1 (46): 66.2 ± 7.4 G2 (46): 67.5 ± 6.7	G1: A + ST + NEST, G2: ST + NEST, F: 7 times/week, D: 4 weeks	RN23, Tunyan (extra), Toupizhen (extra)	VFSS + WST + IFRS	1 WST (G1 < G2) 2 VFSS (G1 > G2) 3 IFRS (G1 > G2)
Zhang et al. [50]	G1 (19): 64.10 ± 8.20 G2 (18): 65.58 ± 10.64	G1: A + rTMS + BT, G2: rTMS + BT, F: 6 times/week, D: 4 weeks	DU20, EX-HN1, ST8, DU16, GB20, RN23, Jialianquan (extra), ST4, ST6, ST7	MBSImP + OTT	G1 < G2 in all outcomes
Zhang et al. [51]	G1 (87): 64.61 ± 9.70 G2 (87): 63.86 ± 10.55	G1: A + ST, G2: ST, F: 3 times/week, D: 8 weeks	DU16, GB20, RN23, Jialianquan (extra), EX-HN15, EX-HN12, EX-HNl3, HT5, LR3, LI4	IFRS + WST	1 WST (G1 < G2) 2 IFRS (G1 > G2)
Zhang and Yin [52]	G1 (62): 70 ± 1 G2 (56): 68 ± 2	G1: A + ST, G2: ST, F: 5 times/week, D: 4 weeks	Shenguan (extra), KI3, LR3	SSA + WST + IFRS	1 WST (G1 < G2) 2 SSA (G1 < G2) 3 IFRS (G1 > G2)
Zhang et al. [53]	G1 (20): 58.3 ± 10.1 G2 (20): 58.2 ± 10.1	G1: A + ST + NEST, G2: ST + NEST, F: 5 times/week, D: 4 weeks	Tunyan (extra), RN23, DU16, SJ17, EX-HN12, EX-HNl3	VFSS + sEMG	1 VFSS (G1 > G2) 2 sEMG (G1 < G2)
Yin et al. [54]	G1 (18): 69.52 ± 6.01 G2 (20): 65.41 ± 7.01	G1: A + ST + NEST, G2: ST + NEST, F: 5 times/week, D: 3 weeks	ST9, RN22, RN23, EX-HN12, EX-HNl3	IFRS + WST	1 WST (G1 < G2) 2 IFRS (G1 > G2)
Gao et al. [55]	G1 (30): 64 ± 5 G2 (30): 65 ± 5	G1: A + BT + ST, G2: BT + ST, F: 5 times/week, D: 4 weeks	DU16, BL10, GB12, RN23, Jialianquan (extra), EX-HN12, EX-HNl3	VFSS + SSA + sEMG	1 VFSS (G1 > G2) 2 sEMG (G1 < G2) 3 SSA (G1 < G2)
Dong [56]	G1 (60): 55.3 ± 6.4 G2 (60): 55.3 ± 6.4	G1: A + ST, G2: ST, F: 5 times/week, D: 2 weeks	EX-HN12, EX-HNl3, DU16, DU15, RN23	VFSS + WST	1 WST (G1 < G2) 2 VFSS (G1 > G2)
Deng et al. [57]	G1 (53): 59.2 ± 11.6 G2 (52): 59.8 ± 13.2	G1: A + ST + NEST, G2: ST + NEST, F: 5 times/week, D: 3 weeks	PC6, DU26, SP6, GB20, SJ17, GB12, Yanhoubi (extra), RN23	WST + SSA	1 WST (G1 < G2) 2 SSA (G1 < G2)
Zhu et al. [58]	G1 (35): 54.97 ± 5.10 G2 (35): 56.26 ± 6.17	G1: A + BT + ST, G2: BT + ST, F: 6 times/week, D: 2 weeks	Yushizhen (extra), LI15, LI11, LI10, SJ5, SJ3, LI4, ST32, GB34, ST36, ST40, GB40, LR3, SP6	IFRS + WST	1 WST (G1 < G2) 2 IFRS (G1 > G2)

G1 > G2/G1 < G2 indicates that the difference between the two groups was statistically significant, *P* < 0.05; G1 = G2 indicates that no significant differences were noted between the two groups, *P* ≥ 0.05. G: group; G1: experimental group; G2: control group; A: acupuncture; ST: swallowing treatment; BT: basic treatment; NEST: neuromuscular electrical stimulation; rTMS: repetitive transcranial magnetic stimulation; F: frequency; D: duration; WST: Watian swallowing test; SSA: standard swallowing assessment; VFSS: videofluoroscopic swallowing study; IFRS: Ichiro Fujishima rating scale; SWAL-QOL: swallow quality-of-life questionnaire; BI: Barthel index; FMA: Fugl–Meyer assessment; CT7R: Caiteng 7 rank; sEMG: surface electromyography; HAMA: Hamilton anxiety scale; HAMD: Hamilton depression scale; RSST: repetitive saliva swallowing test; MBSImP: modified barium swallow impairment profile; OTT: oral transit time.

**Table 2:** The risk of bias assessment.

Reference	Randomization	Allocation concealment	Blinding	Incomplete data	Selective report	Other bias
Wang et al. [24]	Low risk Randomized by random number table	Unclear risk Allocation schedule was not mentioned	Unclear risk Blinding unclear	Low risk None lost to follow-up	Low risk All outcomes reported	Unclear risk
Jiang et al. [25]	Low risk Randomized by random number table	Unclear risk Allocation schedule was not mentioned	Unclear risk Blinding unclear	High risk 10 participants dropout	Low risk All outcomes reported	Unclear risk
Wu et al. [26]	Low risk Randomized by random number table	Unclear risk Allocation schedule was not mentioned	Unclear risk Blinding unclear	Low risk None lost to follow-up	Low risk All outcomes reported	Unclear risk
Wei et al. [27]	Low risk Randomized by random number table	Unclear risk Allocation schedule was not mentioned	Unclear risk Blinding unclear	Low risk None lost to follow-up	Low risk All outcomes reported	Unclear risk
Xia et al. [28]	Low risk Randomized by random number table	Low risk Automated assignment system	Low risk Participants and outcome assessors blinded	High risk 14 participants dropout	Low risk All outcomes reported	Unclear risk
Zeng et al. [29]	Low risk Randomized by random number table	Unclear risk Allocation schedule was not mentioned	Unclear risk Blinding unclear	Low risk None lost to follow-up	Low risk All outcomes reported	Unclear risk
Chang et al. [30]	Low risk Randomized by random number table.	Unclear risk Allocation schedule was not mentioned	Unclear risk Blinding unclear	Low risk None lost to follow-up	Low risk All outcomes reported	Unclear risk
Chen et al. [31]	Low risk Randomized by random number table	Unclear risk Allocation schedule was not mentioned	Unclear risk Blinding unclear	High risk 3 participants dropout	Low risk All outcomes reported	Unclear risk
Feng et al. [32]	Low risk Randomized by random number table	Unclear risk Allocation schedule was not mentioned	Unclear risk Blinding unclear	Low risk None lost to follow-up	Low risk All outcomes reported	Unclear risk
Guo et al. [33]	Low risk Randomized by random number table	Unclear risk Allocation schedule was not mentioned	Unclear risk Blinding unclear	Low risk None lost to follow-up	Low risk All outcomes reported	Unclear risk
He et al. [34]	Low risk Randomized by random number table	Unclear risk Allocation schedule was not mentioned	Unclear risk Blinding unclear	Low risk None lost to follow-up	Low risk All outcomes reported	Unclear risk
Li et al. [35]	Low risk Randomized by random number table	Unclear risk Allocation schedule was not mentioned	Unclear risk Blinding unclear	Low risk None lost to follow-up	Low risk All outcomes reported	Unclear risk
Li et al. [36]	Low risk Randomized by random number table	Unclear risk Allocation schedule was not mentioned	Unclear risk Blinding unclear	Low risk None lost to follow-up	Low risk All outcomes reported	Unclear risk
Li and Gu [37]	Low risk Randomized by random number table	Unclear risk Allocation schedule was not mentioned	Unclear risk Blinding unclear	Low risk None lost to follow-up	Low risk All outcomes reported	Unclear risk
Liu et al. [38]	Low risk Randomized by random number table	Unclear risk Allocation schedule was not mentioned	Unclear risk Blinding unclear	Low risk None lost to follow-up	Low risk All outcomes reported	Unclear risk
Qiao et al. [39]	Low risk Randomized by random number table	Unclear risk Allocation schedule was not mentioned	Unclear risk Blinding unclear	Low risk None lost to follow-up	Low risk All outcomes reported	Unclear risk
Wang et al. [40]	Low risk Randomized by random number table	Unclear risk Allocation schedule was not mentioned	Unclear risk Blinding unclear	High risk 8 participants dropout	Low risk All outcomes reported	Unclear risk
Wang [41]	Low risk Randomized by random number table	Unclear risk Allocation schedule was not mentioned	Unclear risk Blinding unclear	Low risk None lost to follow-up	Low risk All outcomes reported	Unclear risk
Xu [42]	Low risk Randomized by random number table	Unclear risk Allocation schedule was not mentioned	Unclear risk Blinding unclear	Low risk None lost to follow-up	Low risk All outcomes reported	Unclear risk
Yang et al. [43]	Low risk. Randomized by random number table.	Unclear risk. Allocation schedule was not mentioned.	Unclear risk. Blinding unclear.	Low risk. None lost to follow-up	Low risk. All outcomes reported	Unclear risk
Yu et al. [44]	Low risk Randomized by random number table	Unclear risk Allocation schedule was not mentioned	Unclear risk Blinding unclear	Low risk None lost to follow-up	Low risk All outcomes reported	Unclear risk
Zhu [45]	Low risk Randomized by random number table	Unclear risk Allocation schedule was not mentioned	Unclear risk Blinding unclear	Low risk None lost to follow-up	Low risk All outcomes reported	Unclear risk
Zhou et al. [46]	Low risk Randomized by random number table	Unclear risk Allocation schedule was not mentioned	Unclear risk Blinding unclear	Low risk None lost to follow-up	Low risk All outcomes reported	Unclear risk
Zhou et al. [47]	Low risk Randomized by random number table	Unclear risk Allocation schedule was not mentioned	Unclear risk Blinding unclear	Low risk None lost to follow-up	Low risk All outcomes reported	Unclear risk
Zhi et al. [48]	Low risk Randomized by random number table	Unclear risk Allocation schedule was not mentioned	Unclear risk Blinding unclear	Low risk None lost to follow-up	Low risk All outcomes reported	Unclear risk
Zhang and Li [49]	Low risk Randomized by random number table	Unclear risk Allocation schedule was not mentioned	Unclear risk Blinding unclear	Low risk None lost to follow-up	Low risk All outcomes reported	Unclear risk
Zhang et al. [50]	Low risk Randomized by random number table	Unclear risk Allocation schedule was not mentioned	Unclear risk Blinding unclear	Low risk None lost to follow-up	Low risk All outcomes reported	Unclear risk
Zhang et al. [51]	Low risk Randomized by random number table	Unclear risk Allocation schedule was not mentioned	Unclear risk Blinding unclear	Low risk None lost to follow-up	Low risk All outcomes reported	Unclear risk
Zhang and Yin [52]	Low risk Randomized by random number table	Unclear risk Allocation schedule was not mentioned	Unclear risk Blinding unclear	Low risk None lost to follow-up	Low risk All outcomes reported	Unclear risk
Zhang et al. [53]	Low risk Randomized by random number table	Unclear risk Allocation schedule was not mentioned	Unclear risk Blinding unclear	Low risk None lost to follow-up	Low risk All outcomes reported	Unclear risk
Yin et al. [54]	Low risk Randomized by random number table	Unclear risk Allocation schedule was not mentioned	Unclear risk Blinding unclear	Low risk None lost to follow-up	Low risk All outcomes reported	Unclear risk
Gao et al. [55]	Low risk Randomized by random number table	Unclear risk Allocation schedule was not mentioned.	Unclear risk Blinding unclear	Low risk None lost to follow-up	Low risk All outcomes reported	Unclear risk
Dong [56]	Low risk Randomized by random number table	Unclear risk Allocation schedule was not mentioned	Unclear risk Blinding unclear	Low risk None lost to follow-up	Low risk All outcomes reported	Unclear risk
Deng et al. [57]	Low risk Randomized by random number table	Unclear risk Allocation schedule was not mentioned	Unclear risk Blinding unclear	Low risk None lost to follow-up	Low risk All outcomes reported	Unclear risk
Zhu et al. [58]	Low risk Randomized by random number table	Unclear risk Allocation schedule was not mentioned.	Unclear risk Blinding unclear	Low risk None lost to follow-up	Low risk All outcomes reported	Unclear risk
